# Involuntary Twitching of the Head Relieved by Trifacial Neurotomy

**Published:** 1897-07

**Authors:** W. L. Williams

**Affiliations:** Professor of Surgery and Obstetrics, N. Y. State Veterinary College, Ithaca, N. Y.


### INVOLUNTARY TWITCHING OF THE HEAD RELIEVED BY TRIFACIAL NEUROTOMY.

By W. L. Williams, V.S.,
PROFESSOR OF SURGERY AND OBSTETRICS, N. Y. STATE VETERINARY COLLEGE, ITHACA, N. Y.

We occasionally meet with a peculiar disease, habit, or vice in the horse which apparently no one has attempted to describe, though doubtless observed by almost every veterinarian of experience, the chief symptoms of which are involuntary shaking or twitching of the head and rubbing of nose and lips against convenient objects.

In our personal experience the abnormality has been chiefly noted in young horses of marked vigor of constitution, highly fed, and well kept, with but moderate or very light work. It develops gradually and has attained well-fixed characters before the owner has noticed what seems to him a hallucination or vice. The animal more or less frequently gives the head a sudden jerk or shake, as though tormented by insects, though no cause for the movement is discernible. The owner or driver will attribute the difficulty to an irritation of the bit, something about the nose or the ears, but a close study indicates that the movements are not subject to the control of the animal.

The symptoms are in evidence chiefly in winter in mild cases. They disappear with the advent of warm weather, to reappear upon the return of winter, while in severe cases the symptoms are much ameliorated during the warmer months. The symptoms are aggravated when the animal is ridden or driven, but continue in the stall. As the head is twitched there is generally considerable motion of the muzzle, the upper lip being moved as though irritated, and if a convenient obstacle is near the lip is rubbed in a hurried and quite intense way, as though to dislodge an insect.

A close inspection fails to reveal any anatomical lesion to explain the symptoms. Owners have prevailed upon us to clip the hairs from within the concha, under the belief that possibly these irritated the external ear; other owners have applied anodynes to the part. We have examined the teeth in detail, hoping there to discover the cause, and have smoothed these; but the mystery of the abnormality, its cause and nature, remained unsolved, and no guesses served to cure or alleviate the difficulty.

During the college year of 1896–'97 three cases of this kind were presented at our free clinics, two of which were turned away as mysteries after one of them had had his teeth carefully examined and smoothed with the rasp.

The third case presented was a well-bred, vigorous young gelding of fine style and action, but rendered comparatively worthless by the constant twitching of his head whether in harness or stall, accompanied by intense rubbing of the upper lip on the manger, pole or other convenient object.

By wearing a small net, in general character like an ordinary cord fly-net, large enough to cover the nostrils and upper lip, the owner found he would go fairly well in harness, but the presence of the net was sufficient to attract attention and prevented his sale. The owner, a dealer, was consequently anxious to have some remedy applied which would render the animal agreeable to drive and salable. He was fully persuaded that the difficulty lay in an irritation or hyperæsthesia of the upper lip, and when assured that sensation in this could be almost wholly destroyed by sectioning the supermaxillary branch of the trifacial, the owner promptly insisted that the operation be tried, which was accordingly done.

The horse was cast, the hair was shaved from the skin covering the infraorbital foramen, and a solution of cocaine injected upon the nerve. The skin was thoroughly disinfected and an incision about three-quarters of an inch long made directly over the course of the nerve, laying it bare. It was then cut through at the infraorbital foramen, after which a section of the nerve about three-quarters of an inch, was dissected out and incised, the wound dressed with iodoform, sutured with catgut, the operation repeated alike on both sides of the head, the patient released, and placed in a box.

The cocaine failed in its mission, and the operation proved one of the most painful we have ever witnessed. After the operation the symptoms were greatly exaggerated, the animal being quite frantic, throwing his head violently and recoiling suddenly whenever his nose came in contact with the stall, feed-box, or hay-rack. In a few hours he was, with some difficulty, hitched with his mate and driven home, a distance of twenty miles, though found exceedingly difficult to manage, a serious disaster being narrowly averted at one time.

Soon after reaching home the exalted nervousness subsided and marked improvement soon set in, the wounds healed aseptically, and within a few days the patient had recovered from both operation and malady and was driving normally, and continues sound now after about four months.

The results of this operation indicate a peripheral neurosis of the trifacial, and go far to contradict the belief that it is a habit or vice.

The operation itself is exceedingly simple and easy, but it is the most painful in which we have ever engaged, and should be undertaken, it seems to us, only under complete general anæsthesia. With proper precautions aseptic healing should be readily attained.

No attempt should be made to work the animal for some days after treatment. Our success seems to indicate that the operation is a radical cure for the malady and capable of fully restoring the usefulness of this class of animals.

